# Cross-cultural adaptation and validation of the International Knee Documentation Committee Subjective Knee Form in Greek

**DOI:** 10.1007/s10195-015-0362-y

**Published:** 2015-06-21

**Authors:** George A. Koumantakis, Konstantinos Tsoligkas, Antonios Papoutsidakis, Athanasios Ververidis, Georgios I. Drosos

**Affiliations:** Department of Physical Therapy, 401 Army General Hospital of Athens, 1 Panagioti Kanellopoulou Avenue, 11525 Athens, Greece; 2nd Department of Anesthesiology-Pain Unit, ATTIKON University General Hospital, School of Medicine, University of Athens, Athens, Greece; Orthopaedic Surgeon, Rethymno, Greece; Department of Orthopaedic Surgery, University General Hospital of Alexandroupolis, School of Medicine, Democritus University of Thrace, Alexandroupolis, Greece

**Keywords:** International Knee Documentation Committee (IKDC), Greek, Cross-cultural adaptation, Validation, Knee, SF-36

## Abstract

**Background:**

Patient-reported outcomes require validation in a particular language and culture before administration for clinical use.

**Materials and methods:**

A systematic translation of the IKDC Subjective Knee Form was initially tested in 30 patients with various knee pathologies to develop the first Greek version (IKDC/SKF-GR). It was then administered to another 80 patients. The test–retest reliability (*n* = 35) and internal consistency (*n* = 80) were examined. Construct validity was tested by correlating the IKDC/SKF-GR with the SF-36 subscales (*n* = 80) and content validity by measuring floor/ceiling effects. Responsiveness was measured in patients with meniscus pathology (*n* = 24).

**Results:**

Patients filled the form without omissions/questions regarding the phrasing of items. Internal consistency was good (Cronbach’s *α* = 0.87) and test–retest reliability very good (ICC_2,1_ = 0.95, SEM = 4.4 and SDC = 12.2). Correlations with the SF-36 subscales confirmed its construct validity. No floor/ceiling effects were recorded. The effect size was large (ES = 1.26).

**Conclusions:**

The IKDC/SKF-GR has comparable measurement properties to the original form.

**Level of evidence:**

Level II.

## Introduction

Several knee-specific patient-reported outcomes (PROs) have been developed to capture current functional and/or symptom status of patients with various knee conditions [6]. The International Knee Documentation Committee (IKDC) Subjective Knee Form, in particular, monitors symptoms and functional status (both in daily and sports activities) and has been extensively validated in patients with various knee pathologies [13, 14] and meniscus injuries [7]. This form has also been found to have equal or superior measurement properties to other similar measures of knee function in patients with complex knee disorders [1], chondral defects [11], meniscus injury (waiting list and post-surgery) [23], ACL rupture and reconstruction [24].

Translations of the IKDC whole form into other languages (http://www.sportsmed.org/Research/IKDC_Forms/) as well as cross-cultural adaptations of the IKDC Subjective Knee Form in the Italian [20], Dutch [12], Thai [17], Brazilian [19], Chinese [10], Korean [15], Persian [9] and Turkish [3] languages have already been reported.

The purpose of this study was to provide a valid Greek version of the widely used IKDC Subjective Knee Form, to inform future knee-related outcome studies performed in Greek-speaking populations, and to provide a common PRO of knee functional status between populations with a different native language. A systematic cross-cultural adaptation process was followed [2], and an evaluation of the internal consistency, between-day reliability, construct and content validity and responsiveness of this form was performed according to current recommendations of the minimum standards of testing the psychometric properties of PROs [21].

## Materials and methods

### The IKDC Subjective Knee Form

The form consists of 10 items assessing ‘symptoms’, ‘sports activities’ and ‘function’, covering all knee-related injuries. The total score is the sum of the individual item scores and then the score is transformed to a scale ranging from 0 to 100. The total score can be calculated if at least 90 % of the items are completed.

### The IKDC Subjective Knee Form translation and cross-cultural adaptation procedure

The systematic translation and cross-cultural adaptation of the original 2000 version of the IKDC Subjective Knee Form has been conducted according to detailed guidelines [2]. Two separate forward translations from American English to Greek were made by two individuals whose native language was Greek but who were also proficient in English. Discrepancies between the two translations were resolved in a meeting and a synthesis of the two translations resulted in a common translation. Two individuals whose native language was English but were also proficient in Greek acted as back-translators of the common translation in Greek, producing two separate translations. An expert committee of a methodologist, a clinician, a language expert and all translators reviewed all reports and resolved any remaining discrepancies, assuring the semantic, idiomatic, experiential and conceptual equivalence between the two language versions. A pre-final version of the scale was initially administered to 30 patients with various knee pathologies that were referred for physical therapy in our hospital (pre-testing), with content and face validity between source and target versions assured, as all patients completed the IKDC Subjective Knee Form in Greek (IKDC/SKF-GR) without omissions and demonstrated a good understanding of the scale items. Therefore, this version was not modified further and was considered the final version, available for download at: http://www.sportsmed.org/uploadedFiles/Content/Medical_Professionals/Research/Grants/IKDC_Forms/IKDC%20Greek(1).pdf.

### The IKDC Subjective Knee Form further validation procedure

The cross-culturally adapted IKDC/SKF-GR form was administered to 80 consecutive patients, 64 (80 %) of which were male and 16 (20 %) female, between March and August 2010. The patient population tested presented with a variety of knee disorders (Table 1) examined in the orthopaedic clinics of our hospital, and had a mean (SD) age of 35.3 (11.9) years, height of 175.6 (8.7) cm and body mass of 81.0 (12.7) kg.

**Table 1:** Patients’ frequency of knee pathologies (*n* = 80)

Knee pathology	*n* (%)
Injury site	
Right	51 (63.7)
Left	27 (33.7)
Bilateral	2 (2.5)
Diagnosis	
ACL injury	41 (51.2)
ACL/MCL injury	1 (1.2)
ACL/meniscus injury	5 (6.2)
Meniscus injury	24 (30.0)
Chondral injury	5 (6.2)
Osteoarthritis	3 (3.7)
Plica	1 (1.2)

To establish the test–retest reliability over a 2-week interval, the scale was filled in twice by a subgroup of the participants (*n* = 35). Internal consistency was also measured, including data of all participants (*n* = 80). Construct (convergent and divergent) validity was tested by correlating the IKDC/SKF-GR with a generic quality of life scale (SF-36) [25] including all participants (*n* = 80). Content validity was tested by measuring the floor and ceiling effects.

Responsiveness of the scale was tested by administration of the scale in the 24 patients of our sample with meniscus pathology on two occasions: on admission and at a 3-month follow-up at the hospital. All patients, depending on their surgical management, had received written instructions by the hospital physical therapy staff upon discharge, to perform a home rehabilitation program consisting of progressive loading, range of motion and quadriceps strengthening exercises under non-weight-bearing and weight-bearing conditions, and ice application for effusion and pain control [18]. Patients were advised to perform the set program for 20 min, 3 times per week. A diary was kept and returned to the physical therapy department at the 3-month follow-up.

### Statistical analysis

All analyses were performed with the IBM SPSS version 22 statistical package, with a 5 % level of significance. Questionnaire data were initially checked for normality of distribution with the Kolmogorov–Smirnov test and were found normally distributed (*P* > 0.05), therefore parametric statistics were employed.

Reliability was assessed with the intraclass correlation coefficient (ICC), which represents a ratio of the variance of interest over the sum of the variance of interest plus error [8]. A two-way random-effects intraclass correlation coefficient type agreement (ICC_2,1_) was calculated, as systematic differences were considered to be part of the measurement error, supplemented with calculation of the standard error of measurement (SEM) and the smallest detectable change (SDC) [8, 16]. Bland–Altman plots were also constructed to depict in a scatter plot format absolute agreement for test–retest measurements with 95 % limits of agreement (LOA) [16]. Between-day systematic differences were tested with a repeated measures ANOVA. Internal consistency was calculated using the Chronbach *α*, which addresses the homogeneity of the items comprising a questionnaire, with values of 0.70 considered fair, 0.80 good and above 0.90 excellent [4].

Construct validity was assessed by correlating the IKDC/SKF-GR with the subscales of the SF-36 (Pearson correlation coefficient). Convergent validity is the degree of correlation of a particular outcome measure with other measures theoretically predicted to correlate with it; and conversely, divergent validity is the degree to which an outcome measure does not correlate with other measures that it is predicted not to correlate with [13]. The SF-36 consists of 8 domains (physical functioning, PF; role limitation due to physical problems, RP; bodily pain, BP; general health perceptions, GH; vitality, VT; social functioning, SF; role limitation due to emotional problems, RE; and mental health, MH), with each directly transformed into a scale from 0 to 100 (higher scores indicate better health status), to describe patients’ physical and mental states [25]. The sum of PF, RP, BP and GH subscales generates a physical component summary (PCS) score and the sum of the VT, SF, RE and MH generates a mental component summary (MCS) score [25]. For content validity/interpretability, floor effects exist if a proportion of patients report the lowest possible score, whereas ceiling effects exist if a proportion of patients obtain the highest possible score upon the administration of the questionnaire. Floor/ceiling effects of <20 % are considered acceptable [12, 13].

Responsiveness was defined as an indicator of patient-related change over time due to treatment effect [22]. The responsiveness index used was the effect size (ES), expressed as the differences in the means of baseline and 3-month follow-up data, divided by the standard deviation at baseline [14, 22]. A value between 0.20 and 0.50 is considered a small effect, between 0.51 and 0.80 a moderate effect and above 0.80 a large effect [5].

## Results

### Patients

The majority of the patients who participated in our study had an isolated ACL injury (51.2 %) or an isolated meniscus injury (30 %); however, patients with various other knee pathologies were included (Table 1). Most of the patients were also male (80 %).

**Fig. 1:**
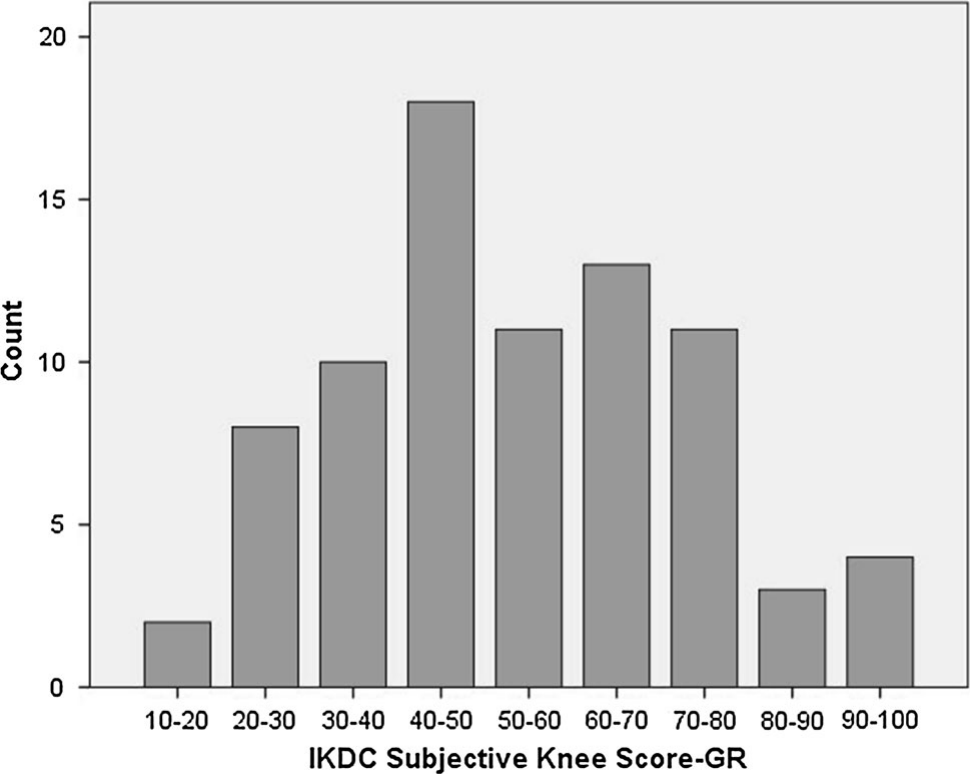
Distribution of calculated International Knee Documentation Committee Subjective Knee Form in Greek (IKDC/SKF-GR) scores (*n* = 80)

The IKDC/SKF-GR was filled in by all patients in approximately 10 min, without omissions, and there were no questions regarding the phrasing of the scale items. The mean (SD) and 95 % confidence interval (CI) data from the IKDC-SKF/GR and the 8 domains and 2 summary scores of the SF-36 questionnaires from all participants are presented in Table 2. The distribution of the IKDC-SKF/GR scores is presented in Fig. 1.

**Table 2:** Mean value, standard deviation, and 95 % confidence interval of the outcome measures (*n* = 80)

	Mean	Standard deviation	95 % Confidence interval
IKDC/SKF-GR	54.21	19.73	49.99–58.59
SF-36 PCS	40.30	10.42	37.95–42.70
SF-36 MCS	48.72	8.47	46.95–50.69
SF-36 PF	62.00	23.82	56.94–67.12
SF-36 RP	30.94	43.07	21.87–41.55
SF-36 BP	56.75	25.93	51.02–62.70
SF-36 GH	74.29	12.41	71.62–77.11
SF-36 VT	63.12	15.41	59.69–66.62
SF-36 SF	65.47	24.78	60.47–71.10
SF-36 RE	53.33	42.63	44.58–63.32
SF-36 MH	73.05	13.76	70.05–76.00

*IKDC*/*SKF*-*GR* International Knee Documentation Committee/Subjective Knee Form in Greek, *SF*-*36* short form 36, *PCS* physical component summary, *MCS* mental component summary, *PF* physical functioning, *RP* role limitation due to physical problems, *BP* bodily pain, *GH* general health, *VT* vitality, *SF* social functioning, *RE* role limitation due to emotional problems, *MH* mental health

### Test–retest reliability and agreement

**Fig. 2:**
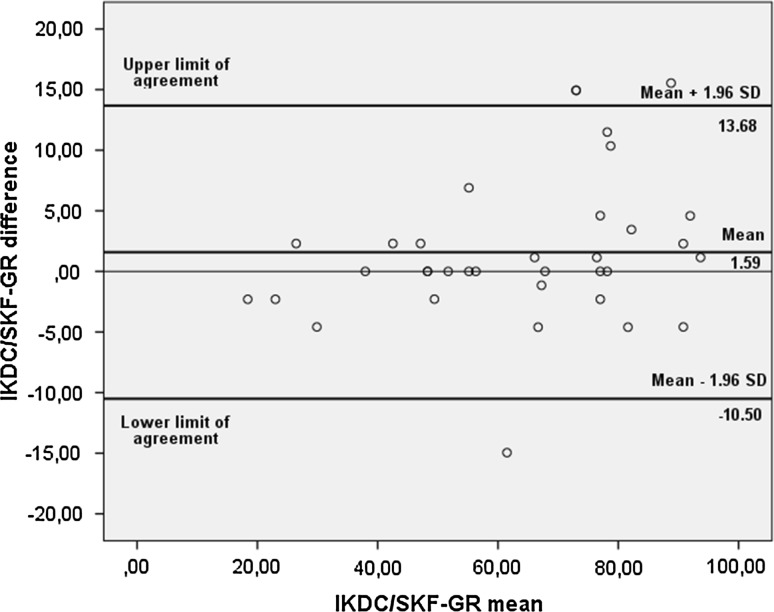
Bland–Altman plots with the 3 *bold lines* representing the ±1.96 SD limits of agreement (*superior* and *inferior*) and the average of the differences (*intermediate*) of the International Knee Documentation Committee Subjective Knee Form in Greek (IKDC/SKF-GR), (*n* = 35)

To assess the test–retest reliability, the form was administered twice (2-week interval) in a group of 35 patients (26 with anterior cruciate ligament reconstruction, 3 with ACL/meniscus, 1 with ACL/MCL, 1 with meniscus, 1 with plica syndrome and 3 with osteoarthritis, of which 31 (88.5 %) were male and 4 (11.5 %) female, with a mean (SD) age of 33.2 (11.6) years, height of 178.6 (5.89) cm and body mass of 83.4 (12.8) kg. Test–retest reliability and agreement indices were considered to have sufficient accuracy and clinical applicability [16], with an ICC_2,1_ (95 % CI) 0.95 (0.91–0.98), SEM = 4.4, SDC = 12.2 and a mean test–retest difference value of 1.59. A repeated measures ANOVA did not demonstrate statistically significant differences between the 2 measurement occasions (*P* = 0.136). The Bland–Altman limits of agreement ranged from −10.50 to 13.68 (Fig. 2).

### Internal consistency

The internal consistency of the IKDC/SKF-GR was good (Cronbach *α* = 0.87).

### Construct validity

The Greek version of the IKDC-SKF demonstrated the strongest correlations with the physical component summary of the SF-36 as well as the physical functioning subscale (*r* = 0.77 for both, *P* < 0.001). Correlations with the bodily pain (*r* = 0.72, *P* < 0.001) and the role limitation due to physical problems (*r* = 0.68, *P* < 0.001) subscales were also strong. The weakest associations were observed between the IKDC-SKF/GR and the mental component summary (*r* = 0.22) as well as the mental health subscale (*r* = 0.26) of the SF-36. Finally, the level of correlation between the IKDC-SKF/GR and the social functioning subscale of the SF-36 (*r* = 0.60, *P* < 0.001), was higher than initially hypothesized (Table 3).

**Table 3:** Pearson correlation coefficients between scores of the IKDC/SKF-GR (*n* = 80), the original and existing cross-cultural adaptations of the IKDC and the SF-36 subscales

	IKDC Subjective Knee Form									
	IKDC/SKF-GR	Original [13]	Italian [20]	Dutch [12]	Thai [17]	Brazilian [19]	Chinese [10]	Korean [15]	Persian [9]	Turkish [3]
SF-36 PCS	0.77**	0.66	0.60	–	0.63	0.79	–	–	0.626	0.70
SF-36 MCS	0.22	0.16	0.40	–	0.34	0.51	–	–	0.159	0.05
SF-36 PF	0.77**	0.63	0.67	0.71	0.75	0.75	0.64	0.66	0.522	0.69
SF-36 RP	0.68**	0.47	0.56	0.55	0.37	0.54	0.50	0.49	0.391	0.53
SF-36 BP	0.72**	0.64	0.75	0.69	0.76	0.63	0.64	0.30	0.679	0.47
SF-36 GH	0.47	0.30	0.26	0.41	0.21	0.54	0.50	0.11	0.336	0.32
SF-36 VT	0.42	0.39	0.36	0.40	0.29	0.46	0.44	0.15	0.402	0.24
SF-36 SF	0.60**	0.47	0.58	0.42	0.22	0.43	0.41	0.48	0.385	0.40
SF-36 RE	0.44	0.26	0.44	0.30	0.34	0.50	0.24	0.30	0.167	0.22
SF-36 MH	0.26	0.25	0.65	0.21	0.29	0.40	0.41	0.15	0.196	0.13

*IKDC/SKF-GR* International Knee Documentation Committee/Subjective Knee Form in Greek, *SF*-*36* short form 36, *PCS* physical component summary, *MCS* mental component summary, *PF* physical functioning, *RP* role limitation due to physical problems, *BP* bodily pain, *GH* general health, *VT* vitality, *SF* social functioning, *RE* role limitation due to emotional problems. *MH* mental health

** Correlation is significant at the 0.001 level (two-tailed)

### Content validity/interpretability

No floor or ceiling effects were identified for the IKDC/SKF-GR, with a minimum value of 18.93 and a maximum value of 93.67 recorded, therefore the content validity/interpretability was good (Fig. 1).

### Responsiveness

Of the 24 patients with meniscus pathology, 6 were only managed conservatively (no surgery), 2 had a meniscus repair and 16 had a partial meniscectomy. Also, 14 (58.3 %) were male and 10 (41.7 %) female, with a mean (SD) age of 39.0 (12.8) years, height of 172.6 (8.51) cm and body mass of 75.4 (10.8) kg. The mean (SD) number of recorded home sessions were 29.6 (4.5) from a maximum of 36 (3 times per week for 12 weeks), indicating a high compliance level, above 80 % of the required. There was no correlation between the number of home sessions performed (ranging between 20 and 36) and the change in the IKDC-SKF/GR recorded.

The IKDC/SKF-GR mean (SD) values on admission to hospital were 49.48 (14.81) and at the 3-month follow-up 68.15 (10.72), recording a mean (SD) increase of 18.67 (7.15) units. The effect size was 1.26, considered to be of a high level.

## Discussion

The process of cross-cultural adaptation of the IKDC-SKF in Greek followed the Guillemin criteria [2] and was subsequently validated in a Greek-speaking population with various knee pathologies, demonstrating comparable measurement properties with the original scale [13, 14] and those in several other languages (Table 3). Specifically, the test–retest reliability and agreement (*n* = 35), the internal consistency, convergent-divergent validity and floor/ceiling effects (*n* = 80), and the responsiveness (*n* = 24) of the IKDC-SKF/GR were examined.

As can be seen in Table 3, for convergent validity, correlation with the PCS, PF and BP subscales of the SF-36 were similar to the original version, while correlation with the RP (*r* = 0.68) and SF (*r* = 0.60) subscales of the SF-36 were higher in our study, compared to the original scale (*r* = 0.47 for both). Divergent validity of the IKDC/SKF-GR was confirmed to be similar to the original version, as correlations with the MCS, GH, VT, RE, MH subscales of the SF-36 were equally low. In addition, no floor or ceiling effects were recorded, which is a desired attribute of a questionnaire for scores not to be clustered at the top or lower end of a questionnaire [21].

The relative reliability index ICC_2,1_ = 0.95 was almost the same as in the original validation paper (ICC = 0.94) [13], as well as in other validation studies. Additional information is contained in the SEM/SDC absolute agreement indices, expressing the degree to which scores are identical, in terms of the original measurement [16]. In our study the SEM = 4.4 and SDC = 12.2 were slightly higher than the original validation of the IKDC/SKF, which reported an SDC of 9 points, with improvements (or deterioration) beyond this range considered as true change. The SDC levels in other validation studies ranged between 6.7 and 16.4 points. In the validation study of the Brazilian version of IKDC, the relevant SEM/SDC values were the lowest (SEM = 2.4/SDC = 6.7) [19], and in the Turkish version the highest (SEM = 6.0/SDC = 16.4) [3], while they were not reported in the other cross-cultural validation studies. In a study performed in patients with isolated meniscus injury the SEM/SDC were found to be similar to the original validation (SEM/SDC = 3.19/8.8) [7]. In another study examining patients with focal articular cartilage defects, the SEM/SDC values were reported to be slightly better in the longer-term than in the shorter-term follow-up (SEM/SDC = 5.6/15.6 in 6 months vs SEM/SDC = 4.9/13.7 in 12 months) [11].

Alternatively, values beyond the limits of agreement, as depicted in the Bland–Altman plots, can be considered as a meaningful change in IKDC scores, signifying an alteration in a patient’s symptomatology [16]. In our study the mean difference between the two testing occasions was 1.59 (not statistically significant), and the LOA was between −10.50 and 13.68. The only other cross-cultural adaptation study reporting LOA values is the Brazilian validation study [19], with a mean difference between testing occasions of only 0.50 and LOAs between −6.1 and 7.1.

The internal consistency of the IKDC/SKF-GR scale was found to be good (*α* = 0.87) and at a slightly lower level than the original scale (*α* = 0.92) [13]. From previous similar studies, the internal consistency Chronbach *α* index ranged between 0.77 and 0.97 [3, 7, 9, 10, 12, 15, 17, 19, 20].

Responsiveness in the original validation of the IKDC in patients with a variety of knee conditions had an ES = 1.13 [14] and in another study involving only patients with meniscus pathology ES = 2.11 [7]. The responsiveness of the IKDC-SKF/GR was also found to be high (ES = 1.26) in our study, tested in a subsample of the whole population used (only in patients with meniscus pathology). Although the IKDC-SKF/GR change score value exceeded the reliability test–retest error (SDC and LOA), only indirect inferences can be made, as the test–retest data were derived from a different subsample, which included only 1 patient with meniscus pathology.

Finally, since the IKDC scale can account for differences across cultures, it may allow for combining and comparing data from populations of different language and cultural backgrounds. Such comparisons may also provide the possibility of studying differences in healthcare delivery and patient management between different countries.

In conclusion, the IKDC Subjective Form in Greek has demonstrated comparable psychometric properties to the original version, therefore the scale is recommended for further use in Greek-speaking patients with knee pathology.
